# Taste disturbance in patients with advanced cancer: a scoping review of clinical features and complications

**DOI:** 10.1007/s00520-023-08012-x

**Published:** 2023-09-06

**Authors:** Marie Hannon, Annelie Shaw, Michael Connolly, Andrew Davies

**Affiliations:** 1 University College Dublin, Dublin, Ireland; 2 Our Lady’s Hospice & Care Services, Dublin, Ireland; 3 Trinity College Dublin, Dublin, Ireland

**Keywords:** Taste, Taste disorders, Neoplasms, Palliative care

## Abstract

**Purpose:**

The purpose of this scoping review is to appraise the published literature on taste disturbance in patients with advanced cancer, with the specific objectives being to determine its prevalence, clinical features and complications.

**Methods:**

This scoping review was conducted using the recommended methodological framework. A detailed search of databases (Medline, Embase, CINAHL and PsycInfo) was conducted to identify eligible studies: eligible studies needed to include patients with advanced cancer and needed to include details of clinical features and/or complications of taste disturbance. Standard bibliographic/systematic review software was used to store the records and manage the review process, respectively.

**Results:**

Twenty-five studies were identified from the database searches. The studies identified included eight physical and/or psychological symptom studies, six symptom cluster studies, five oral symptom studies and six taste and/or smell specific studies. Detailed data is presented on the clinical features and complications of taste disturbance and on the symptom clusters involving taste disturbance in this cohort of patients.

**Conclusion:**

This scoping review identified a relatively small number of relevant studies involving a relatively small number of participants. Nevertheless, it confirms that taste disturbance is a common problem in patients with advanced cancer and is associated with significant morbidity because of the primary condition and the associated complications.

## Introduction

Taste is defined as “the perception derived when chemical molecules stimulate taste receptors in areas of the tongue, soft palate and oropharyngeal region of the oral cavity to perceive the five basic taste qualities: “sweet, sour, salty, bitter and umami” [1]. The terms “taste” and “flavour” are often used interchangeably in real life, clinical practice [2, 3] and the medical literature [4]. However, taste and flavour are distinct entities with flavour encompassing a combination of taste, smell, texture and temperature [5]. Food hedonics encompasses the palatability of foods, where ‘liking’ food is defined as “the immediate experience or anticipation of pleasure from the orosensory stimulation of eating a food” [6]. Taste disturbance can present as a distortion of normal taste sensation (dysgeusia), a reduction in taste sensation (hypogeusia), increased taste sensation (hypergeusia) or as an absence of taste sensation (ageusia) [7].

Taste disturbance can be evaluated subjectively through patient-reported assessments or by objective assessments. In the clinical setting, it can be measured objectively by measuring oral taste sensitivity to tastants through thresholds to some or all the five basic tastes [8]. No independently diagnostic biochemical measures are available [9].

Taste disturbance is relatively common in the general population; data from the US National Health and Nutrition Examination Survey (2013–2014) suggested that the prevalence of objective taste disturbance defined as failing to identify quinine/bitter taste, was 17.3% amongst the US general population [10]. There are a number of different causes for taste disturbance [11], including dental (periodontal) disease, upper respiratory tract infection, medication-related (e.g. antihypertensives, antimicrobials), dental procedure-related trauma, tonsillectomy, middle ear surgery, Bell’s palsy and burning mouth syndrome. Taste and smell disorders often occur simultaneously [9].

Equally, taste disturbance is especially common in patients with cancer [12]. Taste disturbance may occur at diagnosis (treatment naive patients) [13], during anticancer treatment [14], following anticancer treatment (chronic side effect) [15], at disease progression and into cancer survivorship [16]. There are a number of potential causes of taste disturbance in cancer patients, including direct effects of the cancer, indirect effects of the cancer (i.e. paraneoplastic syndromes), adverse effects of anticancer treatments, adverse effects of supportive care measures and co-morbidities (and their management) [17]. It should be noted that the literature already contains a number of reviews relating to taste disturbance in specific subgroups of cancer patients (e.g. lung cancer) [18–20] and specific anticancer treatments (e.g. head and neck radiotherapy) [21].

It has been known for some time that patients with advanced cancer often develop taste disturbances [22]. However, taste disturbance is considered an “orphan symptom”, which is defined as “symptoms not regularly assessed in clinical practice, and consequently little studied and not properly treated” [23]. The aim of this scoping review is to appraise the published literature on taste disturbance in patients with advanced cancer, with the specific objectives being to determine its prevalence, clinical features (i.e. subjective and objective) and impact on the patients (i.e. physical and psycho-social). It appears that there is no analogous scoping review within the clinical literature.

## Methods

This scoping review was conducted using the methodological framework developed by Arksey and O’Malley [24] and incorporating updated guidance on this methodology [25]. The PRISMA Extension for Scoping Reviews (PRISMA-ScR) was used to report the findings [26].

### Search strategy

A detailed search of four electronic databases (Medline, Embase, CINAHL and PsycInfo) was conducted in October 2022. The search strategy for Medline is shown in Appendix. The search strategy was adapted as needed for each electronic database. The search was re-run in January 2023 to check for any new references.

#### Study eligibility criteria

Eligible studies needed to include patients with advanced cancer, as defined by the National Cancer Institute/NCI, USA: “Cancer that is unlikely to be cured or controlled with treatment. The cancer may have spread from where it first started to nearby tissue, lymph nodes, or distant parts of the body” [27]. Studies which included mixed groups of patients with cancer were excluded, unless results for the patients with advanced cancer were separately reported. Studies which focussed on patients with advanced head and neck cancer, and studies that focussed on cancer patients receiving anticancer treatment were excluded. Eligible studies also needed to include details of clinical features and/or complications of taste disturbance. Studies reporting mixed chemosensory changes were excluded, unless results for taste changes were separately reported. Case reports, review articles and other records without original information were not included. Studies involving children (< 19 years) and non-English language studies were excluded.

#### Data management and synthesis

EndNote 20™ bibliographic software (Clarivate Analytics LLP, USA) was used to store the records retrieved from all the searches. Screening of the records was completed using Covidence systematic review software (Veritas Health Innovation, Australia).

Two reviewers (MH and AD) independently screened the titles and abstracts for full text articles to review. A third reviewer (MC) was available to resolve potential conflicts relating to record inclusion. Two reviewers (MH and AS) independently reviewed the full text articles and extracted the relevant information using a review-specific template. A third reviewer (AD) was available to resolve conflicts relating to data extraction.

The reference lists of all retrieved full text articles, pertinent chapters in major palliative care textbooks and pertinent sections of major palliative care guidelines were hand searched for other relevant records. Other sources of relevant records included the researchers themselves.

## Results

### Search results

**Fig. 1:**
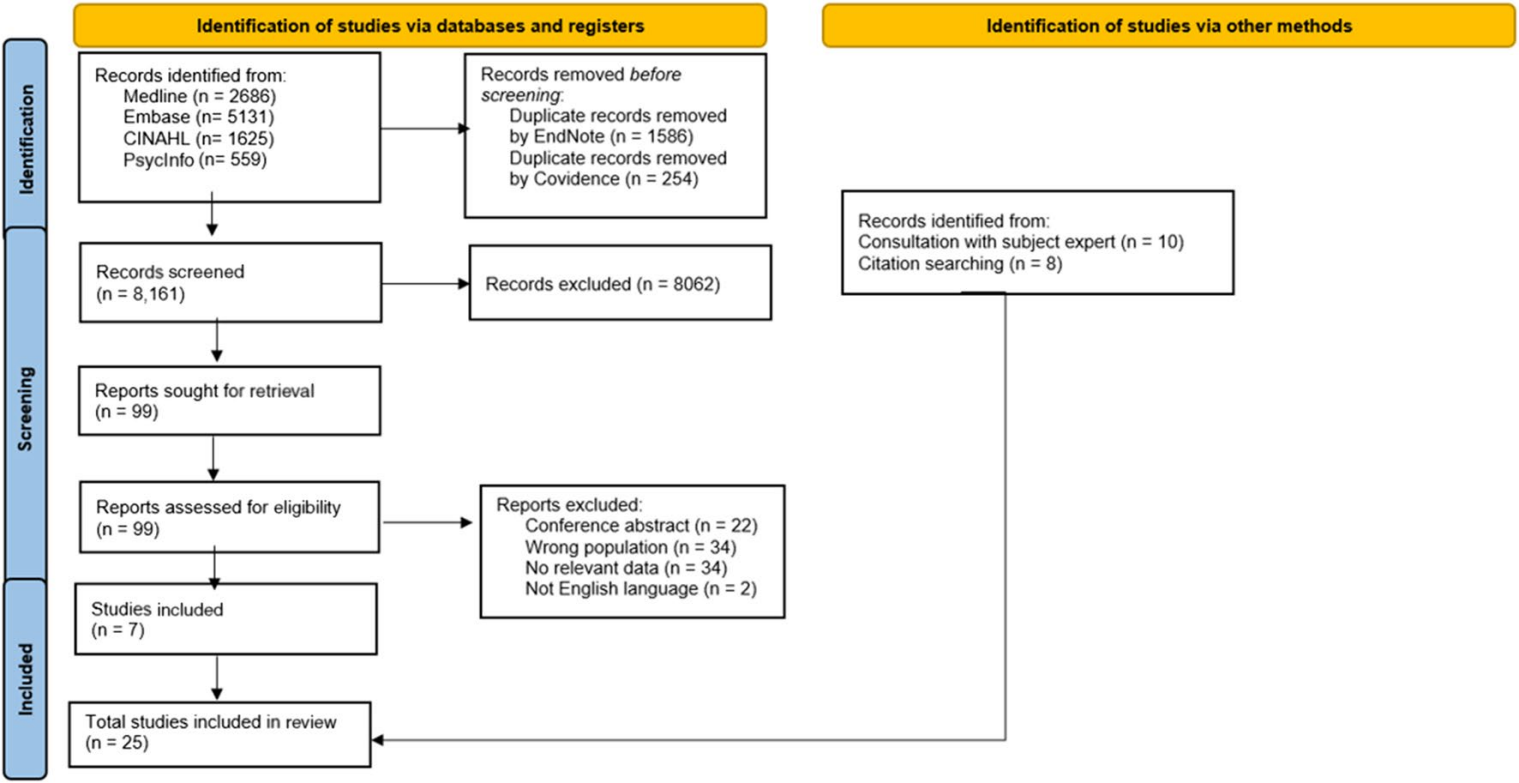
Flow chart of article inclusion

The search strategy identified 8042 references, although only 99 full text articles were retrieved (see Fig. 1). Seven studies were identified from the database searches and had their data extracted [28–34]. Another eighteen studies were identified from handsearching [35–52]. The studies identified included eight physical and/or psychological symptom studies [29, 35, 42, 46, 47, 49, 51, 52], six symptom cluster studies [28, 32, 37, 43, 48, 50], five oral symptom studies [33, 34, 36, 38, 39] and six taste and/or smell specific studies [30, 31, 40, 41, 44, 45] (Tables 1 and 2). Several “duplicate” records were identified amongst the retrieved full text articles; some were conference abstracts, some were journal articles reporting “early” results, and some were journal articles reporting different analyses/subsets of results.

**Table 1:** Clinical features of taste disturbance in patients with advanced cancer

Study	Study population	Methodology	Study results
O’Donoghue et al. (2023) [45]	*n* = 30 Mixed cancer: gastrointestinal (43.5%), lung (30%), urological (10%) Median age: 75 yr (range 46–93 yr) Female: 36.5%	Non-validated questionnaire (modified version of Taste and Smell Survey) [53] Prevalence of “any changes in your sense of taste?”, “persistent bad taste in my mouth” Subjective taste change (bitter, salt, sour and sweet): options — “stronger”, “as strong”, “weaker”, “I cannot taste it at all” Objective taste change (bitter, salt, sour and sweet) using commercial taste strips	Prevalence (subjective taste change): 83% Prevalence (persistent bad taste): 50% Subjective taste change (bitter): “increased perception” — 23%, “decreased perception”— 7% Subjective taste change (salt): “increased perception” — 23%, “decreased perception” — 27% Subjective taste change (sour): “increased perception” — 17%, “decreased perception” — 17% Subjective taste change (sweet): “increased perception” — 27%, “decreased perception” — 17%. Prevalence (objective taste change): 83%, reduced acuity: 16% — one modality undetected; 48% — 2 modalities undetected; 28% — 3 modalities undetected Objective taste change (bitter): incorrect identification — 60% Objective taste change (salt): incorrect identification — 50% Objective taste change (sour): incorrect identification — 63% Objective taste change (sweet): incorrect identification — 20% 60% patients with subjective taste change had objective taste changes: 17% patients had isolated subjective taste change, and 23% patients had isolated objective taste change
Davies et al. (2021) [38]	*n* = 250 Mixed cancer: gastrointestinal (32%), lung (18%), breast (14%) Median age: 68 yr (range 36–91 yr) Female: 58%	Oral Symptom Assessment Scale (OSAS) [38] Prevalence of “taste disturbance” Frequency: options — “rarely”, “occasionally”, “frequently”, “almost constantly” Intensity: options — “slight “moderate”, “severe”, “very severe” Distress: options — “not at all”, “a little bit”, “somewhat”, “quite a bit”, “very much”	Prevalence: 55% Second most common oral symptom reported Frequency: “rarely” — 5%, “occasionally” — 29%, “frequently” — 23%, “almost constantly”— 42% Intensity: “slight” — 23.5%, “moderate” — 45.5%, “severe” — 19.5%, “very severe” — 11.5% Distress: “not at all” — 16.5%, “a little bit” — 30.5%, “somewhat” — 19%, “quite a bit” — 24%, “very much” — 10%
Webber et al. (2021) [51]	*n* = 1507 Mixed cancer: gastrointestinal (52%), urological (12%), lung (10%) Median age: 66 yr (no range) Female: 48%	Memorial Symptom Assessment Scale-Short form (MSAS-SF) [54] Prevalence of “change in the way food tastes” Distress: options — “not at all”, “a little bit”, “somewhat”, “quite a bit”, “very much”.	Prevalence: 56%. Tenth most common symptom reported Distress: “not at all/a little” — 36%, “somewhat” — 21%, “quite a bit/very much” — 43%
McGettigan et al. (2019) [44]	*n* = 30 Mixed cancer: lung (30%), breast (23%), gastrointestinal (13%) Mean age: 68 yr (SD +/− 12 yr) Female: 70%	Non-validated questionnaire (modified version of Taste and Smell Survey) Prevalence of “any changes in your sense of taste?”, “persistent bad taste in my mouth” Frequency of “persistent bad taste in my mouth”: options — “never”, “rarely”, “sometimes”, “often”, “always” Subjective taste change (bitter, salt, sour and sweet): options — “stronger”, “as strong”, “weaker”, “I cannot taste it at all” “Over the past 3 months, I would rate my abnormal taste as”: options — “insignificant”, “mild”, “moderate”, “severe”, “incapacitating” Objective taste change (bitter, salt, sour and sweet) using commercial taste strips	Prevalence (change in sense of taste): 93% Prevalence (persistent bad taste): 60% Frequency (bad taste): “often” — 28%, “always” — 22% Subjective taste change (bitter): “stronger” — 18%, “weaker”/“cannot taste” — 11% Subjective taste change (salt): “stronger” — 21%, “weaker”/“cannot taste” — 32% Subjective taste change (sour): “stronger” — 4%, “weaker”/“cannot taste” — 7% Subjective taste change (sweet): “stronger” — 36%, weaker”/“cannot taste” — 14%. Subjective rating abnormal taste: “insignificant” — 10.5%, “mild” — 29%, “moderate” — 32%, “severe” — 18%, “incapacitating” — 10.5% Prevalence (objective taste change): 53% reduced acuity: 31% — one modality undetected; 50% — 2 modalities undetected; 6% — 3 modalities undetected; 13% — 4 modalities undetected Objective taste change (bitter): reduced acuity — 27% Objective taste change (salt): reduced acuity — 23% Objective taste change (sour): reduced acuity — 37% Objective taste change (sweet): reduced acuity — 20% 43% patients with subjective taste change had objective taste changes, and 100% patients with objective taste changes had subjective taste change
Tebidze et al. (2019) [34]	*n* = 50 Mixed cancer: lung (37%), urological (13%), breast (11%), gynaecological (11%) Age range: 20–75 yr Female: 40%	Non-validated questionnaire Prevalence of “any taste disturbances” Intensity: options — “slight”, “moderate”, “severe”	Prevalence: 70% Intensity: “slight” — 16%, “moderate” — 20%, “severe” — 34%
Van Lancker et al. (2017) [49]	*n* = 400 Mixed cancer: lung (23%), gynaecological (12%), gastrointestinal (10%) Mean age: 75 yr (range 65–93 yr) Female: 48%	Assessment Symptoms Palliative Elderly (ASPE) [55] [ASPE appears to have been modified in this study] Prevalence of “changes in food tasting” Frequency: options — “rarely”, “sometimes”, “often”, “always” “Intensity”: options — “not”, “somewhat”, “moderate”, “very serious”	Prevalence: 35% Frequency: “rarely” — 6%, “sometimes” — 12%, “often” — 13%, “always” — 69% Intensity: “not” 18%, “somewhat” — 12%, “moderate” — 18%, “very serious” — 53%
Alsirafy (2016) [35]	*n* = 89 Mixed cancer: gastrointestinal (26%), central nervous system (21%), lung (14%) Median age: 53 yr (no range) Female — 42%	Open questioning about symptoms/non-validated questionnaire Prevalence of “taste change” Intensity: options — “mild”, “moderate”, “severe”	Prevalence: 27% (questionnaire). No patients reported taste changes on open questioning Intensity: “mild” — 58.5%, “moderate” — 33.5%, “severe” — 8%
Alt-Epping et al. (2012) [36]	*n* = 101 Mixed cancer: gastrointestinal (30%), lung (22%) breast (14%) Age: < 60 yr — 39.5%; ≥ 60 yr — 60.5% Female: 59.5%	Non-validated questionnaire Prevalence of “taste disturbances” Intensity: 0–10 NRS; 0–1 = “quite low”, 4–5 = “moderate”, 9–10 = “quite high”	Prevalence: 67% Intensity: 0–1 — 31%, 2–3 — 7%, 4–5 —34%, 6–8 — 4.5%, 9–10 — 23.5%
Brisbois et al. (2011) [30]	*n* = 192 Mixed cancer: gastrointestinal (32%), lung (25%), breast (19%) (All patients had “a self-perceived taste and/or smell abnormality”) Median age: 64 yr (range 51–76 yr) Female: 49%	Taste and Smell Survey	Prevalence - 86% (26% taste abnormality, 60% taste and smell abnormalities) Subjective taste changes (bitter, salt, sour and sweet) not associated with reduced energy intake
Mahmoud et al. (2011) [31]	*n* = 15 Mixed cancer: lung (26.5%), breast (20%), gynaecological (20%), urological (20%) Median age: 68 yr (range 49–84 yr) Female: 53%	Non-validated questionnaire Prevalence of “food tasted differently” and/or “food preferences changed” Objective taste change (bitter, salt, sour and sweet) using modified Henkin’s 3-drop forced choice test [56]	Prevalence: 80% Subjective taste change: decreased sensitivity to sweet — 40%, decreased sensitivity to salt — 6.5%, “all food tasteless” — 53.5%, “all food bitter” — 20%, “persistent chocolate taste” — 13.5% Objective taste change (salt): increased threshold — 20% Objective taste change (sweet): increased threshold — 26.5% 67% patients with subjective taste change had objective taste changes
Spichiger et al. (2011) [46]	*n* = 103 Mixed cancer: urological (23.5%), gastrointestinal (18.5%), lung (16.5%) Mean age: 63 yr (range 19–89 yr) Female: 38%	Memorial Symptom Assessment Scale (MSAS) [57] Prevalence of “change in the way food tastes” Intensity: options — “slight” (score = 1), “moderate” (score = 2), “severe” (score = 3), “very severe” (score = 4) Distress: options — “not at all” (score = 0), “a little bit” (score = 1), “somewhat” (score = 2), “quite a bit” (score = 3), “very much” (score = 4)	Prevalence: 35% (admission) Intensity: mean score — 2.58/4 Distress: mean score — 2.19/4
Webber et al. (2011) [52]	*n* = 120 Mixed cancer diagnosis: gastrointestinal (28%), breast (14%), lung (13%), urological (13%) Median age: 61 yr (range 20–87 yr) Female: 54%	MSAS-SF (see above)	Prevalence: 62% Distress: “not at all”/“a little bit” — 43%, “somewhat” — 22%, “quite a bit”/“very much” — 35%
Kirkova et al. (2010) [42]	*n* = 181 Mixed cancer: gastrointestinal (29.5%), lung (24%), haematological (9.5%) Mean age: 64 yr (SD +/− 13 yr) Sex: no data	Non-validated questionnaire Prevalence of “taste change” Distress: options — “bothersome/distressful”, “not”	Prevalence: 33% Distress: “bothersome/distressful” — 69%
Bovio et al. (2009) [29]	*n* = 143 Mixed cancer: lung (36.5%), gastrointestinal (33.5%), urological (7%) Mean age: 68 yr (range 57–79 yr) Female: 35%	Non-validated questionnaire (adapted from MSAS-SF) Prevalence of distressing decreased taste acuity (“hypogeusia”), distressing distortion of normal taste (“dysgeusia”) [Symptom deemed present if patient was distressed “somewhat”/“quite a bit”/“very much”, but not “a little bit”]	Prevalence (decreased taste acuity): 22% Prevalence (distortion of normal taste): 23% Decreased taste acuity associated with distortion of normal taste (*p* < 0.001), anorexia (*p* = 0.005), reduced mean energy intake (*p* = 0.014) and BMI (*p* = 0.031) Distortion of normal taste associated with reduced mean energy intake (*p* = 0.005)
Hutton et al. (2007) [41]	*n* = 66 Mixed cancer: breast (29%), gastrointestinal (21%), lung (5%) Mean age: 65 yr (range 53–77 yr) Female: 55%	Taste and Smell Survey Prevalence of “a change in my sense of taste?”, “a food tastes different than it used to”, “a persistent bad taste in mouth” Subjective taste change (bitter, salt, sour and sweet): options — “stronger”, “weaker” “I would rate my abnormal sense of taste as”: options — “insignificant”, “mild to moderate”, “severe to incapacitating”	Prevalence (change in sense of taste): 55% Prevalence (a food tastes different): 47% Prevalence (persistent bad taste): 64% Subjective taste change (bitter): “stronger” — 20%, “weaker” — 3% Subjective taste change (salt): “stronger” — 24%, “weaker” — 17% Subjective taste change (sour): “stronger” — 27%, “weaker” — 3% Subjective taste change (sweet): “stronger” — 27%, weaker” — 12%. Subjective rating abnormal taste: “insignificant” — 48.5%, “mild to moderate” — 42.5%, “severe to incapacitating” — 9% Taste complaint scores (composite scores) associated with reduced energy intake (*p* = 0.018)
Tranmer et al. (2003) [47]	*n* = 66 Patients with “metastatic cancer or stage IV lymphoma” Mean age: 64.14 yr (SD +/− 12.16 yr) Female: 56%	MSAS (see above)	Prevalence: 50% Frequency: “frequently”/“almost constantly” — 76% Intensity: “moderate”/“severe”/“very severe” — 88% Distress: “quite a bit”/“very much” — 45%
Davies (2000) [39]	*n* = 120 Mixed cancer: breast (40%), urological (21%), lung (16.5%) Median age: 66 yr (range 19–89 yr) Female: 61%	MSAS (see above)	Prevalence: 44% Fifteenth most common symptom reported Frequency: “rarely” — 4%, “occasionally” — 20.5%, “frequently” — 40%, “almost constantly” — 35.5% Intensity: “slight” — 30%, “moderate” — 45%, “severe" — 19%, “very severe” — 6% Distress: “not at all” — 17%, “a little bit” — 32%, “somewhat” — 22.5%, “quite a bit” — 21%, “very much” — 7.5% Intensity of taste change associated with intensity of xerostomia (*p* = 0.001)
Davies et al. (1998) [40]	*n* = 112 “Patients with advanced cancer” Age: no data Sex: no data	Non-validated questionnaire Prevalence of “taste problems” Intensity: options — “mild”, “moderate”, “severe” Prevalence of “absence of taste”, “reduction in taste”, “altered taste”	Prevalence: 40% (overall) Prevalence: “absence of taste” — 40%, “reduction in taste” — 31%, “altered taste” — 53%. Intensity: “mild” — 36%, “moderate” — 36%, “severe” — 29%
Sweeney et al. (1998) [33]	*n* = 70 Mixed cancer: incomplete data Mean age: 66 yr (range 42–88 yr) Female: 64%	Non-validated questionnaire Prevalence of “bad or altered taste” Intensity: visual analogue scale (VAS); 0 = “no” problem” to 6 = “severe” problem	Prevalence: 57% Intensity: 4/“moderate” to 6/“severe” — 40%

**Table 2:** Symptom clusters including taste disturbance in patients with advanced cancer

Study	Study population	Study methodology	Symptom cluster																		
			Taste disturbance	Anorexia	Dry mouth	Difficulty swallowing	Fatigue	Early satiety	Weight loss	Constipation	Cough	Mouth sores	“Weakness”	“Lack of energy”	Drowsiness	Dizziness	Shortness of breath	Hair loss	Nausea	Vomiting	Sleep problems
Ozalp et al. (2017) [32]	*n* = 170 Mixed cancer diagnosis Prevalence not stated	Memorial Symptom Assessment Scale/MSAS [57] (“change in the way food tastes”) Hierarchical cluster analysis/HCA — only symptoms with prevalence ≥ 20% included	**x**			**x**					**x**						**x**				
Chaiviboontham et al. (2011) [37]	*n* = 240 Mixed cancer diagnosis 35% had taste disturbance	MSAS (see above) Principal component analysis (PCA) with varimax rotation	**x**			**x**				**x**	**x**	**x**				**x**		x			
Kirkova et al. (2010) [43]	*n* = 181 Mixed cancer diagnosis Prevalence not stated	Non-validated questionnaire (“taste change”) HCA — only symptoms with prevalence ≥ 15% included	**x**	**x**				**x**	**x**												
Tsai et al. (2010) [48]	*n* = 427 Mixed cancer diagnosis 32% had taste disturbance	Non-validated questionnaire (“taste alteration”) Mixed methods (PCA with promax rotation, HCA, K-means cluster method)	**x**	**x**	**x**	**x**				**x**											
Walsh et al. (2006) [50]	*n* = 922 Mixed cancer diagnosis 28% had taste disturbance	Non-validated questionnaire (“taste change”) HCA — only symptoms with prevalence ≥ 15% included	**x**	**x**	**x**		**x**	**x**	**x**				**x**	**x**							
Aktas et al. (2014) [28]	*n* = 922 Mixed cancer diagnosis 27% had taste disturbance Re-analysis of data from Walsh et al. (2006).	Eight different statistical techniques, including repeating the original HCA of symptom prevalence HCA of symptom prevalence, HCA of symptom prevalence at different thresholds and HCA of symptom prevalence with Kappa statistic produced an identical taste-related symptom cluster (see above); the other statistical techniques produced variations on the taste-related symptom cluster (see below)																			
		*HCA of symptom severity with Kendall tau-b*	**x**	**x**	**x**		**x**	**x**	**x**				**x**	**x**					**x**	**x**	**x**
		*K-means cluster method by symptom prevalence*	**x**	**x**	**x**		**x**	**x**	**x**				**x**								
		*K-means cluster method by symptom prevalence with Spearman correlation*	**x**	**x**				**x**	**x**												
		*K-means cluster method of symptom prevalence with Kappa statistic*	**x**	**x**				**x**	**x**												
		*K-means cluster method of symptom severity with Kendall tau-b*	**x**	**x**				**x**	**x**												

### Assessment

The six taste and/or smell specific studies were generally small (median: 48 participants, range: 15–192 participants). All studies involved a subjective assessment, whilst three studies included an objective assessment [31, 44, 45]. Currently, there is no validated tool to assess taste disturbances in this group of patients; four studies [30, 41, 44, 45] utilised the Taste and Smell Survey [53]; and the other studies used non-validated, study-specific questionnaires. Similarly, there is no agreed method for assessing objective taste disturbances in this group of patients (or indeed other groups of patients) [58]; two studies used commercial taste strips [44, 45] and one study used liquid tastants [31].

Importantly, the terminology used in the studies varied, which may have had an effect on the results obtained, especially prevalence statistics: “abnormal taste”, “absence of taste”, “altered taste”, “any taste disturbances”, “bad or altered taste”, “changes in my sense of taste”, “changes in food tasting”, “change in the way food tastes”, “a food tastes different than it used to”, “food tasted differently”, “persistent bad taste in my mouth”, “reduction of taste”, “taste alteration”, “taste change”, “taste disturbance(s)” and “taste problems”. No studies included questions regarding flavour, as opposed to taste. This issue has been highlighted in previous literature reviews involving other cohorts of oncology patients [59].

### Epidemiology

The prevalence of taste disturbance varied widely in the studies included in this scoping review (median: 55%, range: 27–93%) [35, 44]. Alsirafy et al. reported that no patients reported this symptom on open questioning, although 27% patients gave a positive response on systematic assessment (with 41.5% of these patients reporting “moderate”/“severe” intensity) [35]. Importantly, certain studies reported that different assessment tools produced different prevalence figures [38, 44, 45].

Surprisingly, there was little data on factors affecting prevalence (e.g. demographics, cancer diagnosis, performance status and comorbidities). Walsh et al. reported no association between taste disturbance and age, sex or performance status in their patient database [60]. Interestingly, Tranmer et al. reported that taste disturbance was more common in patients with advanced cancer than patients with “end-stage” non-malignant disease [47]. There was limited data on the aetiology of taste disturbance in patients with advanced cancer, although one group of researchers reported an association between the severity of xerostomia (subjective sensation of dry mouth) and the severity of taste disturbance [61]. Furthermore, the same group of researchers reported an oral symptom cluster consisting of xerostomia and taste disturbance (see below) [38].

### Symptom clusters

Table 2 shows studies reporting physical and/or psychological symptom clusters involving taste disturbance [28, 32, 37, 43, 48, 50]. The symptom clusters identified varied from study to study and varied within study (depending on the outcome measure chosen and the statistical method utilised) [28]. It should be noted that there are many other studies reporting physical and/or psychological symptom clusters in patients with advanced cancer, but which did not include the symptom of taste disturbance [62]. Davies et al. (2021) investigated symptom clusters involving specifically oral symptoms and found an association between the presence of taste disturbance and the presence of a “dirty mouth”, and “coating of tongue” (Spearman’s rank correlation coefficient = 0.7) and also an association between the frequency of taste disturbance and the frequency of “dry mouth” (Spearman’s rank correlation coefficient = 0.6) [38]. No analogous studies were identified in the literature.

### Clinical features

Table 1 shows studies reporting the clinical features of taste disturbance [29–31, 33–36, 38–42, 44–47, 49, 51, 52]. It demonstrates that taste disturbance is usually a persistent symptom [38, 39, 44, 47, 49], is often moderate-to-severe in intensity [33–35, 38–40, 46, 47, 49] and is often associated with significant distress [38, 39, 42, 46, 47, 51]. It should be noted that there were many other studies reporting taste disturbance in patients with advanced cancer, but which did not include details about clinical features and/or complications.

Patients reported a variety of subjective taste disturbance, including ageusia [40, 44], hypogeusia [29, 31, 40, 41, 44, 45], hypergeusia [31, 41, 44, 45] and dysgeusia [29–31, 33–36, 38–42, 44–47, 49, 51, 52]. Furthermore, patients often reported more than one type of subjective taste disturbance, and these taste disturbances could affect some or all taste qualities (i.e. bitter, salt, sour and sweet) [31, 40, 41, 44, 45]. Taste disturbance often coexisted with a disturbance in the sense of smell [30, 41, 44, 45].

### Impact of taste disturbances

Taste disturbance may have a major impact on the experience and pleasure associated with eating and drinking, with patients reporting aversions to a variety of foods (e.g. meat) and/or drinks (e.g. alcohol) [31, 63]. Some adopt compensatory strategies such as seasoning food in an attempt to make it palatable [64] or changing their behaviour by ‘taking control’ of when and what they ate [65].

Indeed, taste disturbance may have a major impact on nutritional intake [30, 41] and is one of the preeminent “nutritional impact symptoms” in patients with advanced cancer [38]. Moreover, taste disturbance appears to be a relevant issue in many patients with the cancer-related anorexia/cachexia syndrome [63], with the severity of taste disturbance greater in patients with refractory cachexia, than patients with pre-cachexia, cachexia or without cachexia [66].

Unsurprisingly, taste disturbance may be associated with low mood/depression [64], social isolation (i.e. avoidance of social eating) and an impaired quality of life [30, 41].

## Discussion

This unique scoping review identified a relatively small number of relevant studies involving a relatively small number of participants. Nevertheless, it confirms that taste disturbance is a common problem in patients with advanced cancer and is associated with significant morbidity (because of the primary condition and the associated complications). The results suggest that this so-called “orphan” symptom warrants greater recognition from patients, family carers and especially healthcare professionals.

Importantly, taste disturbance may be amenable to treatment [67] which may result in increased enjoyment of eating and drinking, decreased morbidity and increased quality of life. In addition, resolution/improvement in taste disturbance may prevent/limit malnutrition, which is a major cause of death in this group of patients [68]. Thus, it is important to screen patients for taste disturbance and refer affected patients to an oncology dietician or other appropriate healthcare professional for further assessment/management.

This scoping review has also identified gaps in the current literature and topics for future research in this cohort of patients: (a) observational studies to determine the “risk factors” for taste disturbance (e.g. cancer diagnosis and performance status) — this data would facilitate targeted screening for the problem; (b) observational studies to determine the aetiologies of taste disturbance overall — this data would also facilitate targeted screening for the problem; (c) observational studies to determine the aetiologies of different subtypes of taste disturbance (e.g. ageusia and dysgeusia) — this data would facilitate targeted treatment for the problem; (d) observational studies of patient/family carers unmet needs and priorities for management; (e) development/validation studies of a taste-specific assessment tool for this group of patients — there is a need for a tool that not only assesses the problem but can also assess the response to treatment for the problem, utilising patient-reported outcome measures.

## Appendix. MEDLINE search strategy

1. Taste — MESH
2. Taste alteration — keyword
3. Taste change — keyword
4. Taste disorders — MESH
5. Taste distortion — keyword
6. Taste disturbance — keyword
7. Taste dysfunction — keyword
8. Ageusia — MESH
9. Dysgeusia — MESH
10. Hypergeusia — keyword
11. Hypogeusia — keyword
12. Phantogeusia — keyword
13. Chemosensory problems — keyword
14. Altered taste — keyword
15. Bad taste — keyword
16. Lack of taste — keyword
17. Metallic taste — keyword
18. Taste perception — MESH
19. Taste threshold — MESH
20. Oral symptoms — keyword
21. Oral conditions — keyword
22. Oral diseases — keyword
23. Oral problems — keyword
24. Oral health — MESH
25. Mouth diseases — MESH
26. ***[1 or (2 to 25)]***
27. Neoplasms — MESH
28. Cancer — keyword
29. ***[27 or 28]***
30. ***[26 and 29]***
31. Palliative care — MESH
32. Terminal care — MESH
33. End of life care — keyword
34. ***[31 or 32 or 33]***
35. ***[26 and 34]***
36. ***[35 not 30]***
37. ***[30 or 36]***
